# Spermine modulates fungal morphogenesis and activates plasma membrane H^+^-ATPase during yeast to hyphae transition

**DOI:** 10.1242/bio.029660

**Published:** 2018-01-22

**Authors:** Antônio Jesus Dorighetto Cogo, Keilla dos Reis Dutra Ferreira, Lev A. Okorokov, Alessandro C. Ramos, Arnoldo R. Façanha, Anna L. Okorokova-Façanha

**Affiliations:** 1 Laboratório de Fisiologia e Bioquímica de Microrganismos, Universidade Estadual do Norte Fluminense Darcy Ribeiro, Av. Alberto Lamego, 2000, Pq. Califórnia, Campos dos Goytacazes-RJ 28013-602, Brazil; 2 Laboratório de Biologia Celular e Tecidual, Centro de Biociências e Biotecnologia, Universidade Estadual do Norte Fluminense Darcy Ribeiro, Av. Alberto Lamego, 2000, Pq. Califórnia, Campos dos Goytacazes-RJ 28013-602, Brazil

**Keywords:** Polarized growth, *Yarrowia lipolytica*, H^+^ transport, P-type ATPase, Acid growth theory, Scanning ion-selective electrode technique, Polyamine

## Abstract

Polyamines play a regulatory role in eukaryotic cell growth and morphogenesis. Despite many molecular advances, the underlying mechanism of action remains unclear. Here, we investigate a mechanism by which spermine affects the morphogenesis of a dimorphic fungal model of emerging relevance in plant interactions, *Yarrowia lipolytica*, through the recruitment of a phytohormone-like pathway involving activation of the plasma membrane P-type H^+^-ATPase. Morphological transition was followed microscopically, and the H^+^-ATPase activity was analyzed in isolated membrane vesicles. Proton flux and acidification were directly probed at living cell surfaces by a non-invasive selective ion electrode technique. Spermine and indol-3-acetic acid (IAA) induced the yeast-hypha transition, influencing the colony architecture. Spermine induced H^+^-ATPase activity and H^+^ efflux in living cells correlating with yeast-hypha dynamics. Pharmacological inhibition of spermine and IAA pathways prevented the physio-morphological responses, and indicated that spermine could act upstream of the IAA pathway. This study provides the first compelling evidence on the fungal morphogenesis and colony development as modulated by a spermine-induced acid growth mechanism analogous to that previously postulated for the multicellular growth regulation of plants.

## Abstract (precis)

**Summary:** This study presents a new mechanistic model for the integrative role of the polyamine spermine and hormone auxin in the signaling of yeast-to-hypha transition, filling an important gap in fungal morphogenesis.

## INTRODUCTION

Parallels between the morphogenesis of fungi and plants are worth seeking so that we can make use of the conceptual framework already established with the hope of developing models that can shed new light on the conservative evolutionary mechanisms and influence a myriad of ecological interactions within and among these organisms (Moore, 2013). Fungi are organisms with adaptive morphological plasticity that enables them to survive under challenging conditions and colonize new habitats. The hyphal development and polarized growth contribute to their evolutionary success and are the bases for fungal proliferation and ecological interactions, including virulence and symbiosis with plants (Flor-Parra et al., 2007; Ramos et al., 2008, 2009). Polarized hyphal growth promotes the substrate invasion, directional translocation between host environments, consolidation of the colony, nutrient acquisition, and the formation of 3-dimensional matrices (Sudbery, 2011; Brand, 2012). Many fungi can grow either as unicellular yeast or mycelial forms and can undergo a morphogenesis switch from isotropic to polarized growth (Harris, 2011). Cell wall dynamics have been widely explored in plant as well as fungi cells, and despite their intrinsic differences in components and architecture, cell polarity in plants and fungi involves a common sequence of events underlying the continuous synthesis of proteins, lipids and cell wall building blocks, changes in cytoskeletal dynamics, internal hydrostatic pressure, localized Ca^2+^ gradients, and tip-directed transport of secretory vesicles and accumulation (Lew, 2011; Riquelme, 2013).

The ascomycete *Yarrowia lipolytica* has the ability to grow as yeast, pseudohyphae or true hyphae depending on the environmental conditions and genetic regulatory mechanism (Dominguez et al., 2000). This dimorphic fungus is one of the more intensively studied ‘non-conventional’ species due to its high biotechnological potential and wide range of industrial and environmental applications (Nicaud, 2012; Barth, 2013; Harzevili, 2014; Zinjarde et al., 2014; Ledesma-Amaro and Nicaud, 2016). However, a point so far less explored is that *Y. lipolytica* has also been considered among the beneficial microorganisms in agriculture, proving to be useful as a biofertilizer, associated or not with mycorrhizal fungi, modifying soil physico-chemical, biological and fertility properties that enhance plant performance (Vassilev et al., 2001; Medina et al., 2004; Lonhienne et al., 2014). Ecophysiological roles have also been proposed as the halophyte *Atriplex halimus* was found to interact with halotolerant *Y. lipolytica* strains inhabiting their leaves surfaces (Zvyagilskaya et al., 2001). Moreover, plant-like *HAK* genes encoding Na^+^ transporters were found in *Y. lipolytica*, suggesting that salt adaptive traits in plants and fungi are more extensive than previously thought (Benito et al., 2012).

Many environmental factors, including pH, carbon and nitrogen sources, and oxygen concentrations, are important modulation factors involved in hyphal development and growth (Pérez-Campo and Domínguez, 2001; Ruiz-Herrera and Sentandreu, 2002; Bellou et al., 2014). Microarray and proteomic analysis of *Y. lipolytica* during the yeast-to-hypha transition revealed several genes and proteins involved in morphogenetic transition (Morín et al., 2007; Morales-Vargas et al., 2012). The complete genome sequence and efficient genetic tools have also provided important insights on signaling pathways and transcriptional factors required for morphogenesis in *Y. lipolytica* (Cervantes-Chávez et al., 2009; Martinez-Vazquez et al., 2013). In addition, this non-pathogenic fungus has interesting similarities to the highly virulent pathogen *Candida albicans* (Herrero et al., 1999). In this way, *Y. lipolytica* has emerged as an excellent yeast model to study the mechanisms that drive the morphogenetic transition in fungi (Dominguez et al., 2000; Herrero et al., 1999).

Studies on cell differentiation have demonstrated that polyamines play a key role in hyphae and colony growth and development of many fungal systems (San-Blas et al., 1997; Ueno et al., 2004; 31. Valdés-Santiago et al., 2010; Kummasook et al., 2013). In *Y. lipolytica,* the intracellular levels of polyamines increase before the morphogenetic transition and differentiation process (Guevara-Olvera et al., 1993), but the underlying mechanism is not yet fully understood. Polyamines are low molecular weight positively charged aliphatic molecules that facilitate interactions with macromolecules, stabilizing DNA, RNA, proteins and phospholipids, and modulating gene expression, enzyme activities, and DNA-protein interactions (Tabor and Tabor, 1985). In addition to the morphogenetic transition, the fungi polyamines have also been correlated with cell cycle progression (Chattopadhyay et al., 2002), defense against reactive oxygen species (Chattopadhyay et al., 2006), and cell lifespan (Eisenberg et al., 2009, 2016). The differential polycationic character of putrescine, spermidine and spermine (Spm), have been related to the distinct properties and functions of each polyamines (Tabor and Tabor, 1985).

P-type plasma membrane H^+^-ATPase plays an essential role in fungal and plant cells physiology. This proton pump generates the electrochemical proton-motive force across the membrane that drives the energy-dependent uptake of amino acids, sugars, nucleosides, and inorganic ions (Goffeau and Slayman, 1981). In addition, H^+^ transport mediated by this enzyme contributes to the regulation of intracellular pH and surface pH along the hyphae. In plants, it is widely accepted that the activation of plasma membrane H^+^-ATPase by indole-3-acetic acid (IAA) underlies the induction of polarized growth of roots and pollen tube expansion (Hager, 2003; Zandonadi et al., 2010; Takahashi et al., 2012). This mechanism is the base of the classical acid growth theory, which postulates that the activation of proton pump by auxin and subsequent pH decrease in the apoplast promotes plant cell growth (Hager et al., 1991; Rayle and Cleland, 1992; Frías et al., 1996). Interestingly, although the presence of IAA in fungi has long been reported (Roberts and Roberts, 1939; Gruen, 1959), it was just recently that a role for auxin has been related to the morphological transition in *Saccharomyces cerevisiae*, stimulating the morphogenetic switch from yeast cells to a pseudohyphal form (Prusty et al., 2004), and to the hyphal growth in the human pathogen *C. albicans* (Rao et al., 2010).

A transmembrane pH and electrical gradient might be critical in establishing the cell polarity and regulating the assembly of cytoskeletal components required for hyphal extension (Harold, 1990). A transient increase in the intracellular pH was reported before the morphogenetic transition in *C. albicans* (Stewart et al., 1988), as well as at the extending hyphal tip in *Neurospora crassa* (Robson et al., 1996). In fact, P-type H^+^-ATPase is rate-limiting for growth and the decrease of ATPase activity correlates with decreased intracellular pH in yeast cells (Portillo and Serrano, 1989). Moreover, extracellular neutral or alkaline pH induces hyphal development in *Y. lipolytica* and *C. albicans,* revealing the importance of the H^+^ gradient to hyphal morphogenesis (Ruiz-Herrera and Sentandreu, 2002; Vylkova et al., 2011). Thus, fungal and plant cells share similar features in ion homeostasis and cellular bioenergetics that might be involved in the modulation of polarized cell growth.

Although there is a body of evidence suggesting that pH is an essential factor in fungal morphogenesis and that P-type H^+^-ATPase regulates the membrane microenvironment pH in these organisms, the actual role of this pump in polarized hyphal growth remains elusive. Moreover, it has been shown that Spm can modulate IAA-dependent P-type H^+^-ATPase activation and cell polarized growth in plants (Garufi et al., 2007; Pandolfi et al., 2010; Dutra et al., 2013; Pottosin et al., 2014); however, to date, no study has explored whether Spm could play a role in the modulation of P-type H^+^-ATPase during polarized growth in fungi. Therefore, the present work aims to investigate whether Spm modulates the morphogenesis and polarized cell growth of the model fungus *Y. lipolytica* through mechanisms similar to those found in plants, underlying an activation of plasma membrane H^+^-ATPase and the recruitment of auxin-dependent pathways.

## RESULTS

### Spermine induces *Y. lipolytica* filamentous growth

**Fig. 1.**
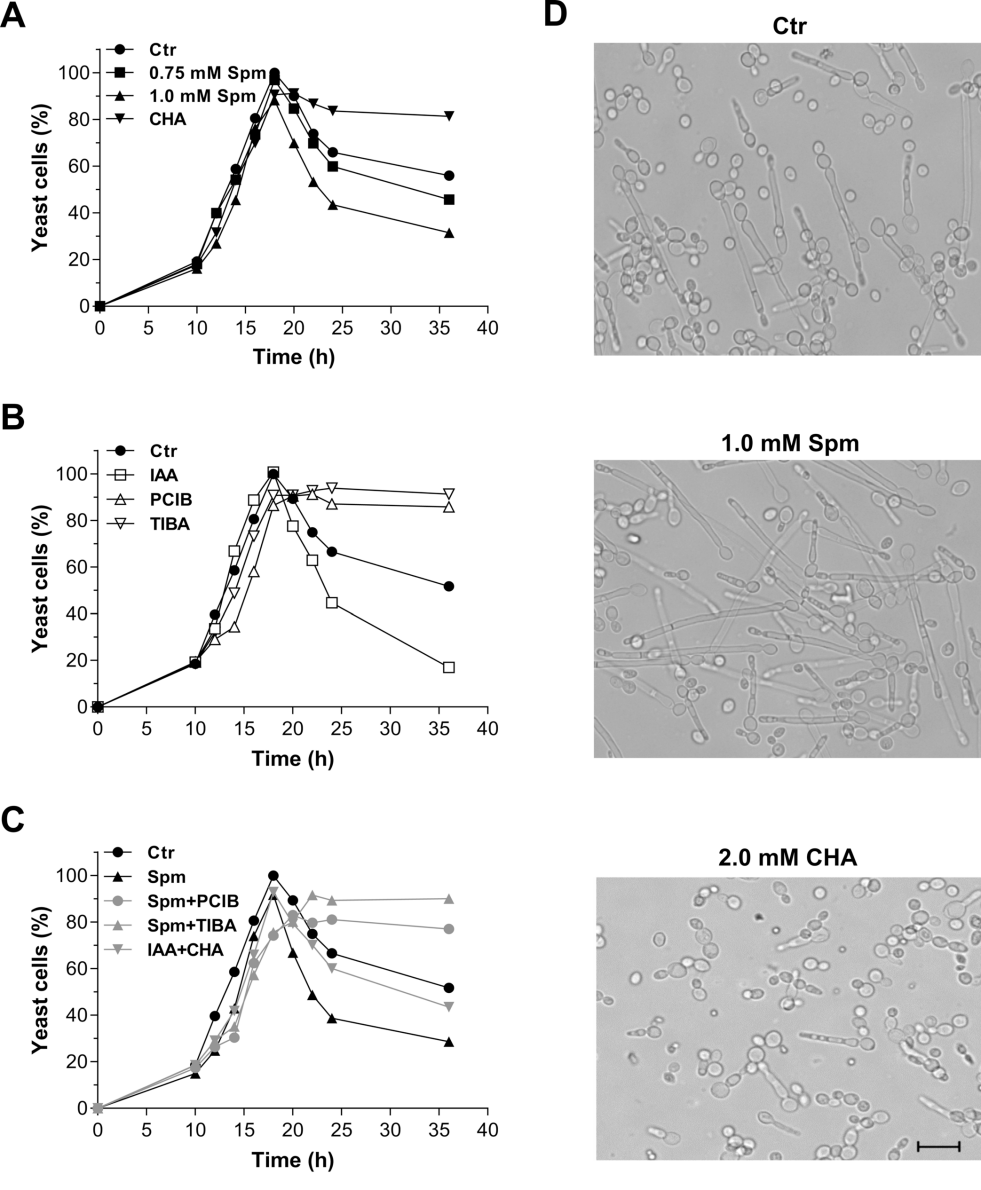
**Effect of Spm on *Y. lipolytica* cell morphology**. (A-C) *Y. lipolytica* cells were grown in YED medium supplemented with Spm at the indicated concentrations, 10 pM IAA and their inhibitors (2 mM CHA, 100 µM PCIB, 100 µM TIBA). The number of yeast cells at each time point was counted and the maximal value was considered as 100%. Values are representative of at least three independent experiments. (D) Visualization of the Spm-dependent effect on the morphogenic transition of *Y. lipolytica.* Cell morphology was examined in cultures grown for 36 h in YED medium supplemented or not with Spm or CHA. Scale bar: 20 µm. Ctr, control; Spm, spermine; CHA, spermidine synthase competitive inhibitor cyclohexylamine; IAA, indole-3-acetic acid; PCIB and TIBA, auxin inhibitors.

*Yarrowia lipolytica* cells grown in liquid YED medium reached the stationary phase after 22 h. Microscopic analysis of cell morphology revealed that the morphogenetic transition took place after 18 h of growth. Consistent with this observation, the number of yeast cells declined after this time point due to the increase of pseudohyphae and hyphae forms (Fig. 1A). Different concentrations of Spm (0.1-2 mM) affected neither the growth nor the morphogenesis start point, although concentrations higher than 1.5 mM Spm reduced cellular growth and caused the appearance of abnormal cells (data not shown).

Spermine concentrations between 0.75 and 1.5 mM potentiated the dimorphic transition in *Y. lipolytica* with the highest effectiveness found at 1 mM Spm. At this concentration of Spm, 70-80% of the *Y. lipolytica* culture was in the yeast form after 20 h of growth, whereas the corresponding values for the control culture ranged from 85-90%. After 36 h, only 30-35% of cells cultured in the presence of Spm remained in the yeast form, whereas yeast cells made up 50-55% of the control culture (Fig. 1A,D). Furthermore, most of the filamentous forms observed in the cells treated with Spm were true hyphae (Fig. 1D). Additionally, the presence of 2 mM CHA, a competitive spermidine synthase inhibitor, reduced significantly (*P*≤0.05) the morphogenesis, resulting in 80% of the cells remaining in the yeast form even after 36 h (Fig. 1A,D), although it did not interfere with cell growth. These results indicate that Spm is required for *Y. lipolytica* morphogenetic transition.

To investigate a putative signaling pathway involving Spm in the modulation of hyphal growth, we cultivated cells in the presence of 1 mM Spm concomitantly or not with an inhibitor of auxin signaling [α-*p*-chlorophenoxyisobutyric acid (PCIB), 100 µM] or inhibitor of auxin transport [2,3,5-triiodobenzoic acid (TIBA), 100 µM]. At these concentrations, the inhibitors impaired the morphogenetic transition in control culture and did not affect cell growth. Treatments involving addition of PCIB and TIBA had a similar effect to that of CHA, and maintained the yeast form by nearly 80-90% of cells. Addition of low concentrations of IAA (10 pM) induced the morphogenetic transition (Fig. 1B) and reverted the inhibitory effect of CHA (Fig. 1C). On the other hand, the inhibitory effect of TIBA and PCIB was not antagonized by Spm (Fig. 1C). The data suggest that modulation of morphogenetic transition by Spm might involve IAA signaling.

**Fig. 2.**
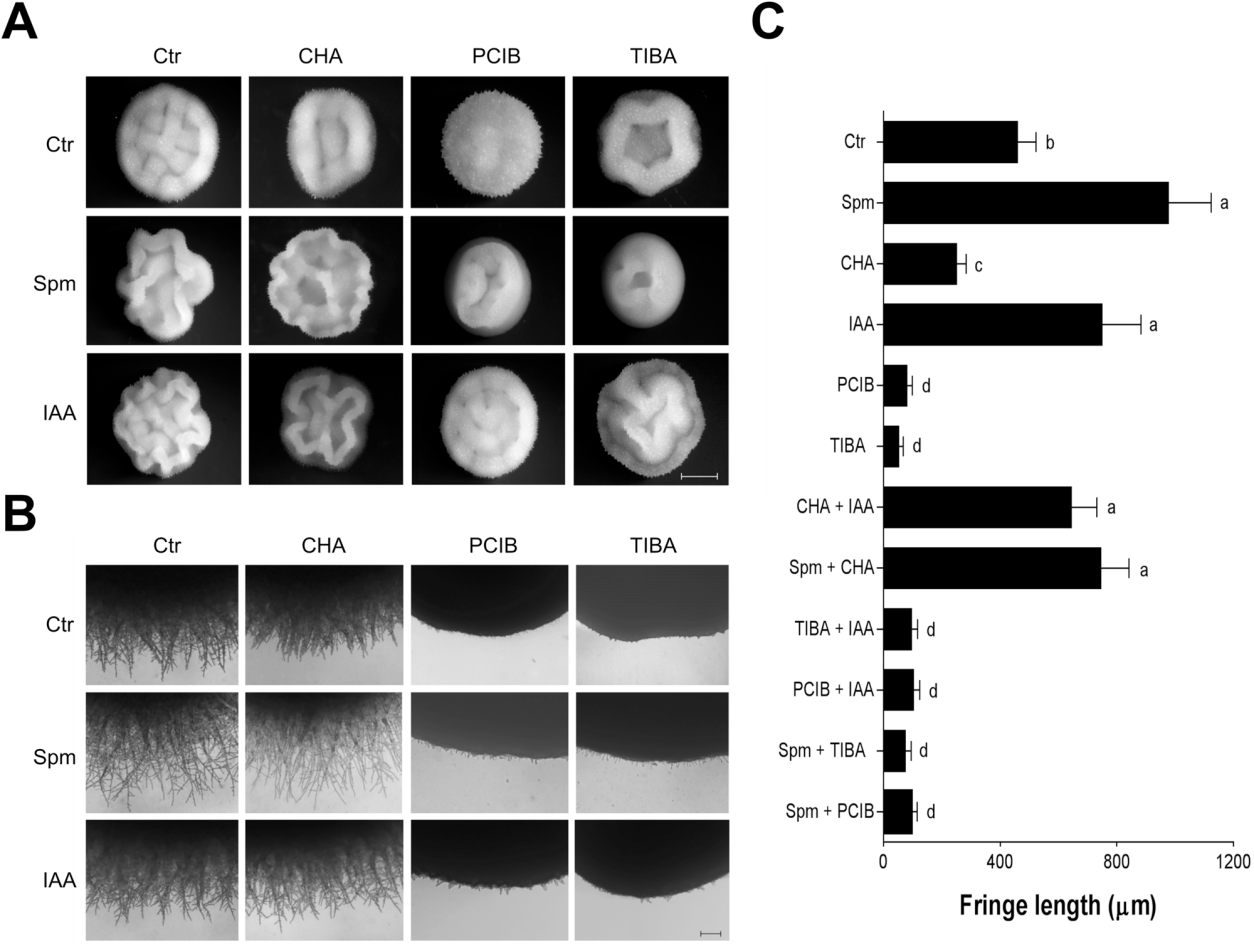
**Effect of Spm on *Y. lipolytica* colony morphology**. (A) Colony morphology of *Y. lipolytica.* Cells were plated onto YED medium supplemented with 1 mM Spm and 10 pM IAA and their inhibitors (2 mM CHA, 100 µM PCIB, 100 µM TIBA), incubated for 4 days at 30°C, and photographed using a Zeiss Stereo Discover V8 stereomicroscope equipped with an Axiocam MRc5 digital camera. Representative micrographs from three independent experiments are shown. Scale bar: 2000 µm. (B) Borders of the colonies grown as described above were documented on a Zeiss Axio Observer A.1 inverted microscope equipped with a digital camera. Representative micrographs from three independent experiments are shown. Scale bar: 200 µm. (C) The fringe length after 4 days of growth. Values are mean±s.d. (*n*=20). Differences between the means were analyzed by one-way ANOVA followed by Tukey test. Different letters represent significant differences by Tukey test (*P*≤0.05). For abbreviations see Fig. 1.

The morphology of *Y. lipolytica* colonies was examined on solid medium (Fig. 2). Colonies of control cells displayed a central interlaced patch surrounded by short peripheral extensions or fringes (460±64 µm). In contrast, the presence of 2 mM CHA resulted in ring-like colonies containing much shorter fringes (250±33 µm). *Y. lipolytica* cells grown on Spm-containing plates formed irregular wrinkled colonies. Fringes developed in the presence of 1 mM Spm were distinctly longer, reaching nearly twice the length of those observed in control colonies (976±147 µm). The addition of 10 pM IAA also resulted in highly structured wrinkled colonies with long fringes (749±133 µm), while the auxin inhibitors PCIB or TIBA caused nearly smooth colony phenotype with almost no surrounding extensions. It is of note that IAA overcame the effect of CHA and increased the extent of colony wrinkling, as well as restored the fringe length (Fig. 2A,C).

**Fig. 3.**
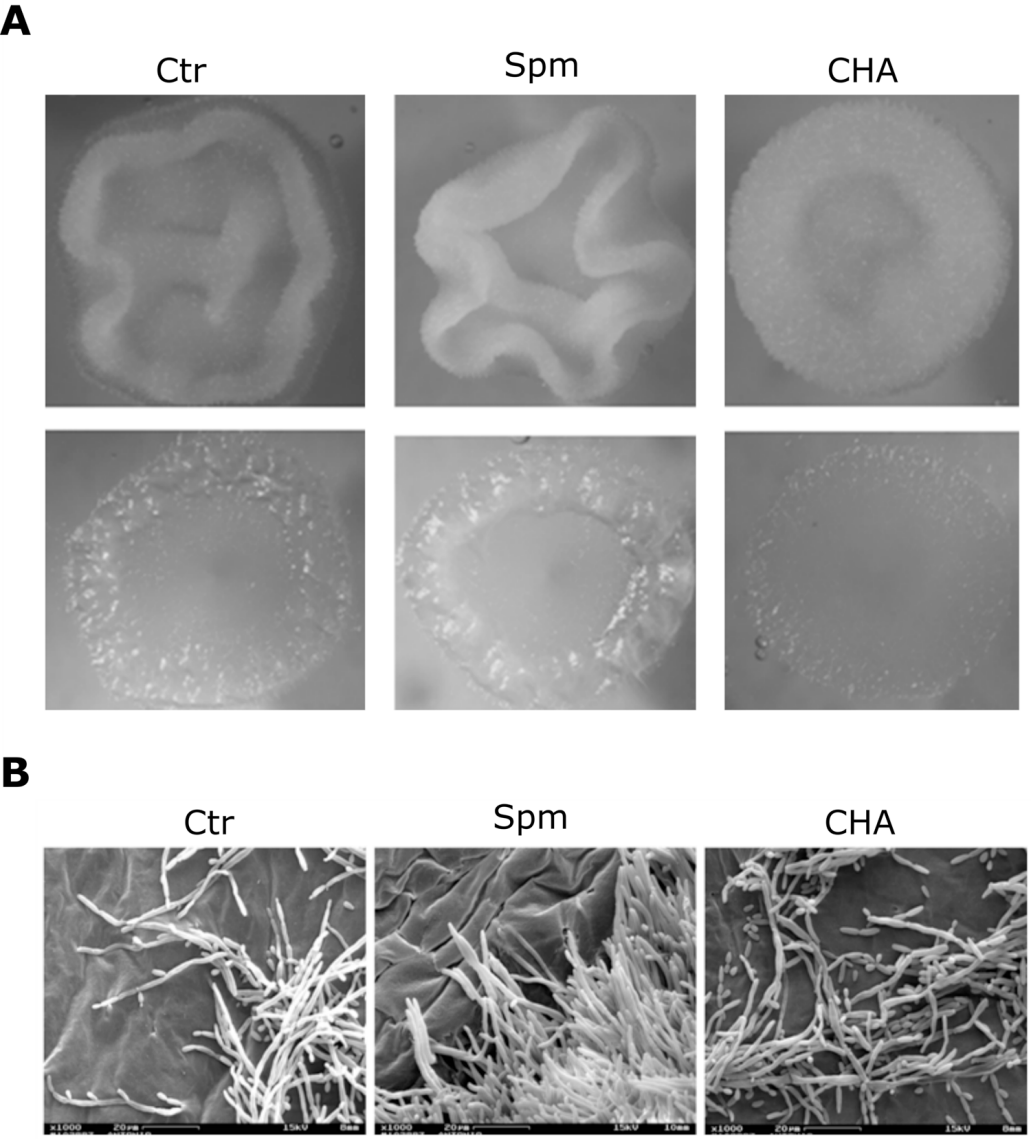
**Spm induces *Y. lipolytica* filamentation and invasive growth**. (A) Plate-washing assay. Cells were plated onto YED medium with or without 1 mM Spm and 2 mM CHA. The plates were incubated for 4 days at 30°C to allow the formation of colonies. The plates were photographed before (upper image) and after (lower image) washing cells off the agar surface. (B) Scanning electron micrographs of borders of the colonies after 4 days of growth on YED plates in the presence or absence of 1 mM Spm and 2 mM CHA as indicated. Scale bars: 20 µm. Representative micrographs are shown.

The ability of Spm to induce filamentous growth was further analyzed. Microscopic examination revealed invasive scars patterns by *Y. lipolytica* colonies; the extent of agar invasion was enhanced in the presence of 1 mM Spm and abolished by 2 mM CHA (Fig. 3A). Scanning electron microscopy of *Y. lipolytica* colonies illustrates in detail that colony morphology is closely associated with *Y. lipolytica* cell morphotypes; less convoluted colonies formed in the presence of the inhibitor CHA were composed mainly of yeast and pseudohyphal cells, whereas wrinkled colonies on Spm-containing plates were composed mainly of invasive hyphal cells (Fig. 3B).

### Modulation of morphogenesis by spermine involves the production of IAA

**Fig. 4.**
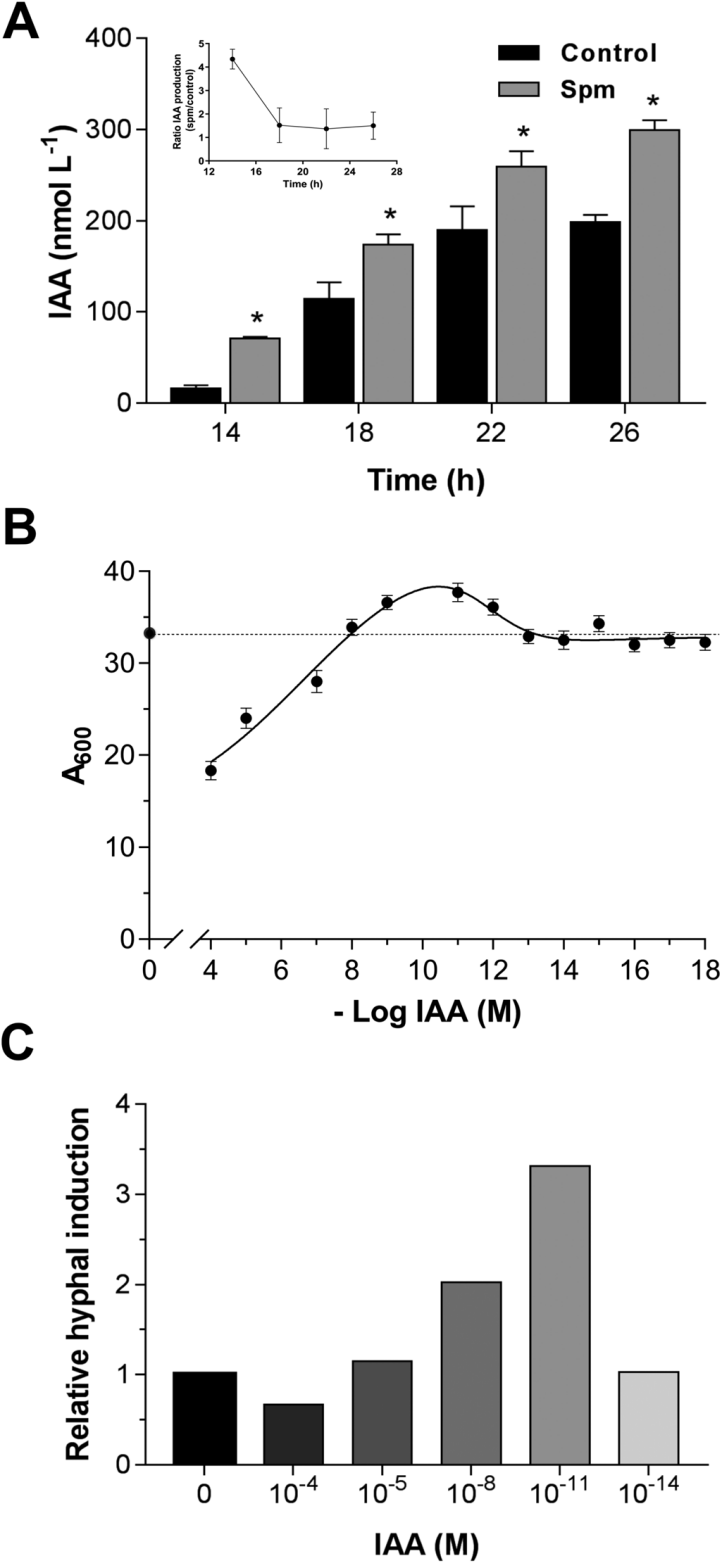
**Spermine and IAA-related effect on *Y. lipolytica*.** (A) Spm induces IAA production. Extracellular concentrations of IAA after 10, 14, 18, 22 and 26 h of *Y. lipolytica* growth in YED medium with or without 1 mM Spm. Inset: the ratio of IAA levels between Spm-treated and untreated cells. Values are means±s.d. (*n*=3). Differences between the means were analyzed by one-way ANOVA followed by Tukey test (*P≤0.05). The effects of exogenous IAA on *Y. lipolytica* growth (B) and morphogenesis (C) were analyzed after 22 h and 36 h, respectively, in liquid YED cultures. In B, values are mean±s.d. (*n*=3); in C, values represent the relative hyphal induction normalized to hyphal induction in control (IAA-untreated) cells (*n*=3).

In addition to the above described stimulatory effect of Spm on morphogenesis and the activation of P-type H^+^-ATPase, *Y. lipolytica* cells grown in the presence of Spm increased significantly the content of IAA in the extracellular media, when compared with untreated cells (Fig. 4A). Interestingly, the data revealed a significant difference in IAA content between treated and untreated cells mainly at the pre-transition stage, suggesting that the synthesis of this molecule by yeast cells might be also related to yeast-hypha transition. The levels of detected IAA were ranging from 15 to 300 nM (Fig. 4A); therefore, we examined the effect of different exogenous IAA concentrations (from 10^−4^ to 10^−14^ M) on *Y. lipolytica* growth and morphogenesis. Additions of low concentrations of IAA had slight effect on growth while supplementation with 10 or 100 µM IAA promoted inhibition of growth (Fig. 4B). Remarkably, low concentrations such as 10 nM and 10 pM induced morphogenesis and hyphal formation (Fig. 4C); the filamentous forms in auxin-containing medium were more pronounced than that of the control and a dominance of hyphae was observed in stationary 36-h culture. At high concentrations of IAA (100 µM) the dimorphic switching was delayed (Fig. 4C), and some pseudohyphal/hyphal forms appeared only after 41 h of cultivation (data not shown). These results suggest that IAA can cause both stimulatory and inhibitory effects on *Y. lipolytica* depending on the concentration, and that IAA can induce filamentation at physiological pM-nM range compatible with amount of IAA produced by cells.

### Spermine stimulates the plasma membrane H^+^-ATPase

**Fig. 5.**
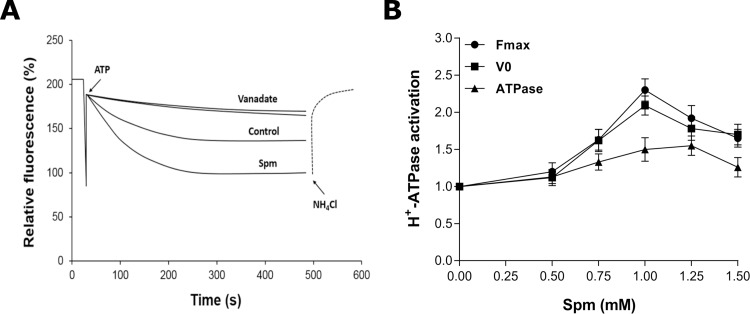
**Spm activates plasma membrane *P*-type H^+^-ATPase.** (A) ATP-dependent and vanadate-sensitive formation of ΔpH across the total membranes isolated from *Y. lipolytica* cells grown for 20 h in YED medium with or without 1 mM Spm. H^+^ transport was initiated by the addition of 1 mM ATP; the proton gradient was dissipated by 20 mM NH_4_Cl. H^+^ transport was sensitive to 0.2 mM vanadate. Data shown are representative of at least three independent membrane isolation experiments. (B) Concentration-dependent activation of the steady-state (*F_max_*) and initial velocity (*V*_0_) of H^+^ transport, and ATP hydrolysis by Spm. *Y. lipolytica* cells were grown for 20 h in liquid YED medium containing the indicated Spm concentrations and used for membrane vesicles isolation as described in the Materials and Methods. Values are means±s.d. of three independent experiments.

The P-type plasma membrane H^+^-ATPase activity was investigated as a potential target of Spm. First, we demonstrated that 0.2 mM sodium orthovanadate, a specific inhibitor of P-type ATPase, prevented 85-90% of the ACMA fluorescence quenching corresponding to the ATP-dependent H^+^ pumping of total membranes vesicles isolated from *Y. lipolytica* cells cultivated in the presence or absence of Spm (Fig. 5A). *Y. lipolytica* cells grown in YED media containing different Spm concentrations for 20 h (morphogenetic transition) exhibited activation of the P-type H^+^-ATPase, with 1 mM Spm being the most effective. This concentration increased by ∼2.3-fold the amplitude of the H^+^ pumping (*F*_max_) and by ∼2-fold the initial velocity of H^+^ transport (*V*_0_), whereas ATP hydrolysis was increased by ∼1.5-fold (Fig. 5B).

**Fig. 6.**
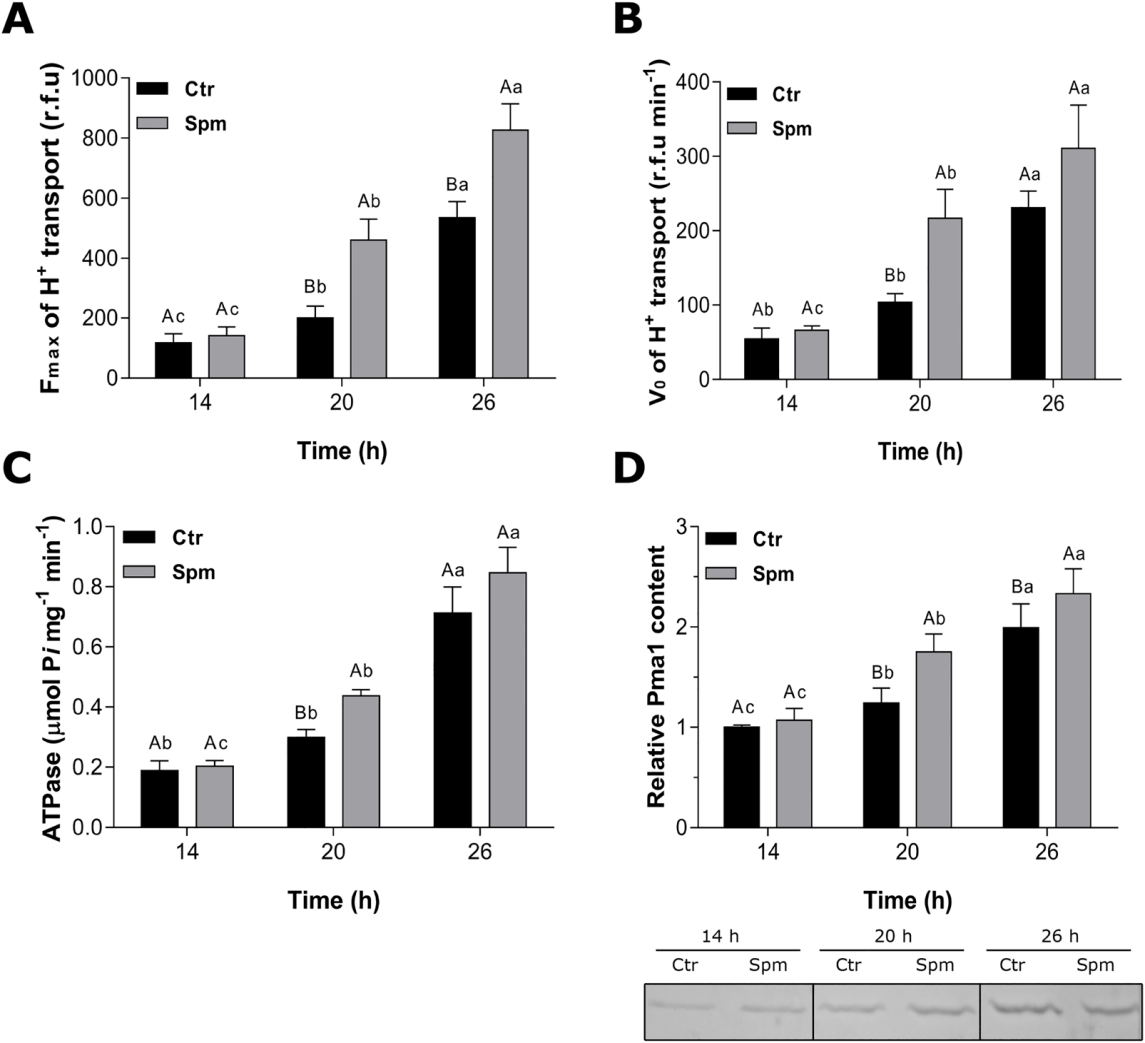
**Modulation of the activity and expression of P-type H^+^-ATPase by Spm during *Y. lipolytica* morphogenesis**. Steady-state (A) and initial velocity (B) of H^+^ pumping, ATP hydrolysis (C) and immuno-response of Pma1p (D) in total membrane vesicles isolated from *Y. lipolytica* cells grown for 14, 20 or 26 h in YED medium supplemented or not with 1 mM Spm. Values are means±s.d. of at least four independent experiments. Differences between the means were analyzed by one-way ANOVA followed by Tukey test. For each point time, means followed by the same uppercase letter are not significantly different by Tukey test (*P*≤0.05); for each treatment, means followed by the same lowercase letter, at different time point, are not significantly different (*P*≤0.05) (*n*=4).

The effect of 1 mM Spm was also analyzed during the *Y. lipolytica* morphogenesis transition. For this, membrane vesicles were isolated at different time points: from yeast cells at pre-transition (14 h), from cells undergoing yeast-to-hypha transition (20 h), and at late stage of hyphal growth (26 h). Proton transport activity in membranes vesicles of control cells was lowest in the yeast form (14 h), and was enhanced by ∼1.5-fold at the yeast-to-hypha transition (20 h) and by ∼4.5-fold in hyphae (26 h). Notably, the H^+^ pumping activity in Spm-treated cells increased by ∼3.3- and 6-fold at 20 and 26 h, respectively, when compared with its 14 h level (Fig. 6A). The initial velocity (*V*_0_) of H^+^ pumping in control cells increased ∼2- and 4-fold after 20 and 26 h of growth, respectively, whereas for Spm-treated cells the induction was ∼3.3- and 4.7-fold (Fig. 6B). The vanadate-sensitive ATP hydrolysis activity of P-type H^+^-ATPase also exhibited a continuous increase accompanying *Y. lipolytica* filamentation, and exhibited a similar degree of Spm induction (Fig. 6C).

To verify if the increase in P-type H^+^-ATPase activity occurred via regulation of proton pump expression or via enzyme post-translational modification, we performed western blot analysis using specific anti-Pma1p antibodies against P-type plasma membrane H^+^-ATPase. We found that the amount of immuno-reactive protein in total membranes from *Y. lipolytica* was higher after yeast-to-hypha transition. Compared to pre-transition values, Pma1p levels in control cells were elevated ∼1.2- and 2.0-fold at 20 h and 26 h of growth, respectively, whereas the corresponding elevations for Spm-cultured cells were 1.6- and 2.2-fold (Fig. 6D). It is of note that Spm further induced Pma1p levels ∼1.5-fold when compared to control cells at 20 h of growth. No difference was observed in immune response between Spm-treated and control cells at the 14 h, just as observed for the H^+^ pumping.

**Fig. 7.**
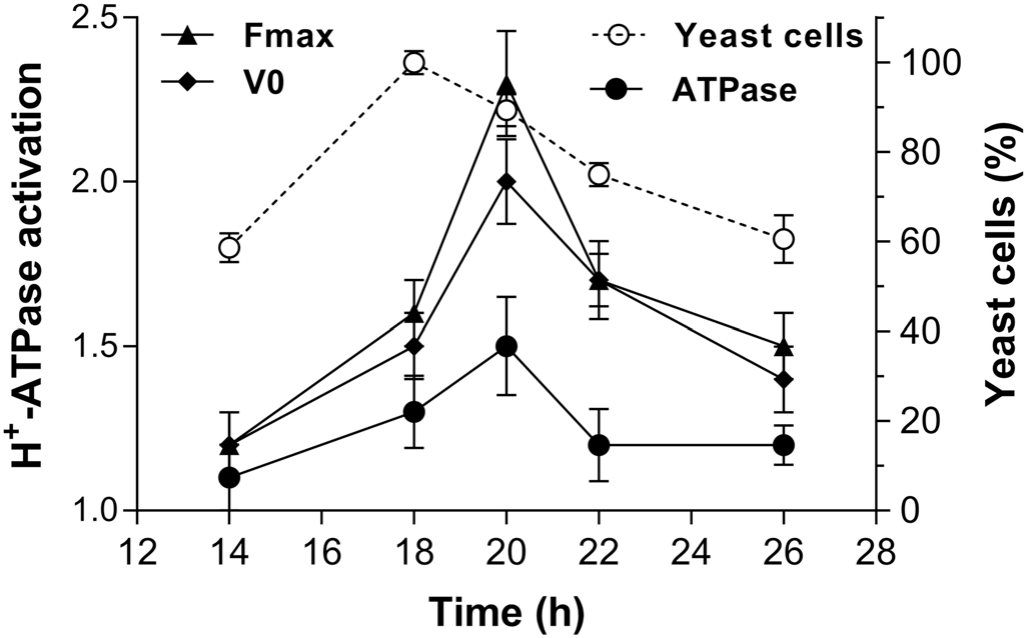
**Correlation between Spm-induced P-type H^+^-ATPase activity and *Y. lipolytica* morphogenesis.** Ratio of steady-state (*F_max_*) and initial velocity (*V_0_*) of H^+^ transport, and ATP hydrolysis between Spm-grown and control cells is shown. Cells were cultivated for 14, 18, 20, 22 or 26 h in YED medium with or without 1 mM Spm. *Y. lipolytica* morphogenesis is plotted as the percentage of yeast cells (see Fig. 1). The transition took place after 18 h. Values are means±s.d. of at least four independent experiments.

The data revealed that the morphogenetic transition of *Y. lipolytica* has a positive correlation with the activation of the P-type H^+^-ATPase. The H^+^ pumping, initial velocity (*V*_0_) of H^+^ transport, ATP hydrolysis and the Pma1p levels increased along with dimorphic transition in cells treated with or without Spm (Fig. 7). Moreover, the confluence at the same time point revealed no difference between control and Spm-treated cells at the pre-transition stage (14 h) for the H^+^ pumping, V_0_, and ATP hydrolysis. During the next hours of growth (18 h), the enzyme exhibited a gradual increase in its activity, and at 20 h reached the maximum activation by Spm for the H^+^ pumping (∼2.3-fold), *V_0_* (∼2.0-fold), and ATP hydrolysis (∼1.5-fold). At this time point, Spm exerted a higher effect on the H^+^ pumping activity than on ATP hydrolysis, revealing a higher coupling of the pump (higher H^+^ transported/ATP hydrolyzed ratio) as induced by Spm at the transition point. The stimulatory effect by Spm remained after yeast-hypha transition; however, there was a decline in difference between control and Spm-treated cells (Fig. 7). Altogether, these data clearly indicate that Spm can potentiate the *Y. lipolytica* morphogenesis in coordination with the induction of P-type H^+^-ATPase activity.

### Spermine induces the H^+^ flux of *Y. lipolytica* cells

**Fig. 8.**
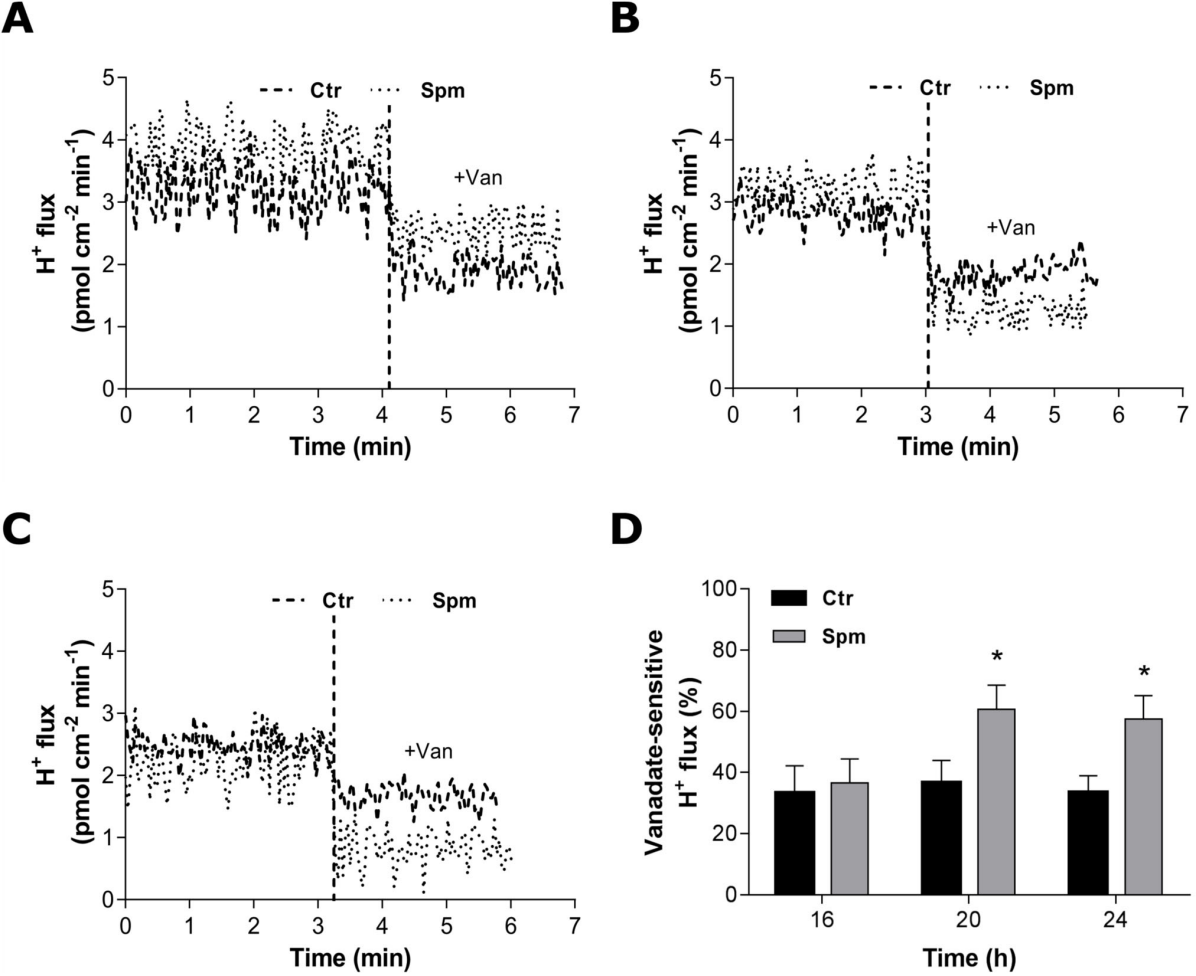
**Temporal profile of extracellular H^+^ efflux in *Y. lipolytica* cells**. (A-C) Proton flux over time was measured using non-invasive SIET in live cells after 16 h (A), 20 h (B) and 24 h (C) of growth in YED medium with or without 1 mM Spm. Vanadate (1 mM Na_3_VO_4_) was added to cells after stabilization of H^+^ efflux. One representative profile of five independent experiments is shown in each case. (D) Comparison of vanadate-sensitive H^+^ efflux at different morphological stages. Values are means±s.d. (*n*=5). Differences between the means were analyzed by one-way ANOVA followed by Tukey test (*P≤0.05, between H^+^ efflux in the control and Spm-grown cells). Ctr, control; Spm, spermine.

We also examined the H^+^ flux at the surface of *Y. lipolytica* live cells at different morphological stages using the non-invasive scanning ion-selective electrode technique. H^+^ flux was measured in yeast cells at pre-transition (16 h), in cells undergoing yeast-to-hypha transition (20 h) and in filamentous cells (24 h). H^+^ efflux was detected in all these conditions (Fig. 8). Control and Spm-grown pre-transition yeast cells exhibited H^+^ efflux of similar magnitude (3.18±0.39 pmol cm^−2^ min^−1^ and 3.86±0.36 pmol cm^−2^ min^−1^, respectively). Direct addition of vanadate to the cells resulted in a decrease in H^+^ efflux (Fig. 8A), revealing the participation of the plasma membrane P-type H^+^-ATPase in H^+^ flux. Similar profiles of H^+^ efflux were obtained for the cells at two other morphogenetic stages (Fig. 8B and C, respectively). However, the contribution of vanadate-sensitive component was nearly 1.5-fold higher in Spm-grown cells at and after the morphogenetic transition (20 h and 24 h, Fig. 8D). These data highlight a key role of plasma membrane H^+^ pump in Spm-induced H^+^ efflux associated with Spm-stimulated morphogenesis.

## DISCUSSION

Fungi and plants are distinct kingdoms of eukaryotic organisms which have separated in evolution at some level long before the multicellular level of organization was established (Moore, 2013). A highly diverse group of key ecologic importance, fungi remain vastly understudied compared to plants. Fungal dimorphism is a complex phenomenon triggered by many environmental factors and consists of a reversible alternating pattern of growth between different elliptical yeast and filamentous forms of cells. Understanding the mechanisms that regulate these events is of major interest because of their implications in fungal pathogenesis and cell differentiation (Harris, 2011; Mayer et al., 2013). It has been observed that polyamines modulate plant and fungal morphogenesis, but the precise mechanisms underlying polyamine action remain unclear. Here, we investigated this issue by focusing on previously known clues regarding similarities that the plants and fungi share in the polarized cell growth. First, fungi together with plants possess P-type H^+^-ATPase that generates a H^+^ electrochemical gradient across the plasma membrane, which is used by ion and metabolite secondary transporters, and is essential for pH control (Goffeau and Slayman, 1981; Palmgren and Nissen, 2011; Falhof et al., 2016). Second, modulation of polarized growth in plants involves an activation of the P-type plasma membrane H^+^-ATPase through an auxin-dependent pathway (Hager et al., 1991; Rayle and Cleland, 1992). Third, the hyphal growth is characterized by a transcellular ionic current with attendant electric field and a gradient of pH along the hyphae that promote ion uptake and turgor pressure for cellular expansion (Lew, 2011; Harold, 1990). We hypothesized that since fungi and plants possess P-type H^+^-ATPase and a cell wall with complex dynamics, it might be possible that fungi, like plants, activate this pump to modulate polarized growth by polyamines.

In the present study, we have investigated the effect of Spm on *Y. lipolytica* morphogenesis. Among polyamines, Spm is the most charged polyamine and, consequently, more effective to promote interaction with other molecules or proteins, including a potentiated effect when compared with putrescine and spermidine on the P-type H^+^-ATPase activities and H^+^ flux in plant cells (Garufi et al., 2007; Pandolfi et al., 2010; Pottosin et al., 2014). We demonstrated a role for Spm in the regulation of filamentous growth, the effect of Spm on cell morphology, colony morphology of *Y. lipolytica* and substrate invasiveness (Figs 1–3). Furthermore, Spm caused the enhancement of the H^+^ pumping and ATP hydrolytic activity mediated by the plasma membrane P-type H^+^-ATPase (Fig. 5). The concentration of 1 mM Spm was more effective, although lower concentrations also induced yeast-to-hypha transition and the enzyme activity (Figs 1 and 5). Concentrations higher than 1.5 mM Spm caused the appearance of abnormal cells, that is in agreement with previous study reporting the toxicity of high Spm concentrations for microorganisms (Tabor and Tabor, 1985).

The increase of intracellular polyamines content, including Spm, was detected during filamentous growth of *Y. lipolytica* (Guevara-Olvera et al., 1993), as well as 2.6-fold upregulation of spermidine synthase gene expression during yeast-to-hypha transition (Morales-Vargas et al., 2012). The addition of CHA, a competitive inhibitor of the spermidine synthase enzyme, is known to block the intracellular synthesis of spermidine and, consequently, of Spm (Kumar et al., 2011). The effect of CHA confirmed the importance of Spm for *Y. lipolytica* filamentation since CHA strongly inhibited yeast-hypha transition and interfered with colony morphology (Figs 1–3).

Our data suggest that induction of hyphal growth by Spm might be related to stimulation of the P-type H^+^-ATPase. We show that Spm significantly enhanced the vanadate-sensitive H^+^-transport and ATP hydrolysis mediated by plasma membrane H^+^-ATPase, as well as the content of Pma1p during *Y. lipolytica* morphogenesis (Fig. 6). These results are consistent with the observation that hyphae are electrically polarized (Harold, 1990), and the hyphal growth occurs after a transient rise of the intracellular pH (Stewart et al., 1988; Robson et al., 1996). The P-type H^+^-ATPase activity establishes a putative transmembrane H^+^ current, an electric field and membrane potentials that could orient the cytoskeleton and cell polarization (Minc and Chang, 2010; Campetelli et al., 2012; Chang and Minc, 2014) . The stimulatory effect of Spm was also related to the H^+^ transport in plants cells (Pandolfi et al., 2010; Dutra et al., 2013; Pottosin et al., 2014), including a dual capacity of Spm on membrane potential: a hyperpolarization at low concentration and depolarization at higher concentration, possibly by the P-type H^+^-ATPase activity (Pottosin et al., 2014). Importantly, Spm was the only polyamine that increased significantly the hydrolytic activity and the immunoreactivity of P-type H^+^-ATPase in plants cells (Garufi et al., 2007). The present data on Spm effects on the fungal proton pump revealed a possible influence of Spm on the coupling of this enzyme due to stronger increase of H^+^ pumping as compared to the ATPase activity and protein content (Fig. 6). Furthermore, the P-type H^+^-ATPase stimulation by Spm was synchronized with the *Y. lipolytica* morphogenesis (Fig. 7), pointing at the important role of the proton pump in the polarized growth of hyphal cells.

A possible mechanism of proton pump modulation by Spm in plants cells was proposed based on an increase of interaction of 14-3-3 proteins with carboxy-terminal phosphorylated domain of P-type H^+^-ATPase (Garufi et al., 2007). However, the carboxy-terminal domain of fungal proton pump is shorter than in plants cells, its phosphorylation sites are different and thus its regulation may not involve 14-3-3 proteins (Kühlbrandt, 2004). On the other hand, 14-3-3 proteins bind to a wide variety of proteins in yeast cells, function as regulators of enzyme activity, and localization anchors, adapters or scaffolds for many cellular processes (van Heusden and Steensma, 2006). The *Y. lipolytica* genome encodes two 14-3-3 proteins (YlBMH1 and YlBMH2) and the yeast-to-hypha transition is related to the increase in YlBMH1 expression (Hurtado and Rachubinski, 2002). Thus, Spm could promote the stability of proteins and phospholipids membranes, modulating gene expression, and enzyme activities that support the P-type H^+^-ATPase activity in fungal as well as in plant cells. The present data will prompt further investigations regarding H^+^ pump regulation.

Expansion of plants cells also underlies polarized growth mechanistically described by the acid growth theory, which postulates that the growth hormone auxin promotes the cell wall acidification, enhancing the P-type H^+^-ATPase activities and content in the plasma membrane. The acid pH stimulates the cell wall loosening enzymes and initiates the expansion of the cells (Hager, 2003; Rayle and Cleland, 1992; Niczyj et al., 2016). Furthermore, IAA, the most studied auxin, is synthesized by microorganisms using pathways quite similar to that described in plants, including a tryptophan-independent pathway (Rao et al., 2010), and is also related with yeast morphogenesis (Prusty et al., 2004; Rao et al., 2010). Indeed, some hydrolytic enzymes involved in fungal cell wall remodeling exhibit optimum pH at ∼5.0-5.5 (Notario, 1982; Hartland et al., 1996; Fontaine et al., 1997). Therefore, we investigated the relationship between Spm and IAA during *Y. lipolytica* morphogenesis. We found that addition of Spm to growth medium increased significantly the extracellular content of IAA (up to 300 nM), mainly before the transition point (Fig. 4A), and that nM-pM of IAA induced hyphal formation (Fig. 4C).

The interaction of signaling pathways between auxin and polyamines in the modulation of hyphal growth was pharmacologically investigated using two IAA inhibitors with different mode of action, TIBA and PCIB, and the use of CHA, an inhibitor of spermidine synthase. PCIB impairs the plant auxin-signaling pathway by regulating Aux/IAA protein stability (Oono et al., 2003; Biswas et al., 2007), while TIBA impairs IAA transport and P-type H^+^-ATPase vesicle trafficking (Geldner et al., 2001; Soeno et al., 2010). Our data showed that both IAA inhibitors blocked yeast-to-hypha transition and the formation of colonial peripheral extensions, even in *Y. lipolytica* cells grown with Spm (Figs 1 and 2), thus indicating that polyamines and IAA act in the same signaling pathway. In plants cells, there is a cross-regulatory interaction between polyamines and auxin during primary and lateral root development (Hausman et al., 1995; Tonon et al., 2001; Saini et al., 2013). Global changes in genes expression resulted from perturbations of Spm levels were examined by transcript profiling and showed that a decrease in Spm levels in mutant Arabidopsis plants led to downregulation of the auxin transporters, whereas the increase of Spm upregulated multiple auxin-responsive proteins (Gonzalez et al., 2011). Higher Spm and spermidine intracellular levels also enhanced the expression of several auxin-regulated genes in tomato fruit (Kolotilin et al., 2011). Although some experimental data point to a cross-regulatory interactions between polyamines and auxin in plant cells, further characterization of the role of polyamines in regulating auxin functions is required to elucidate the role of polyamines in the auxin signaling (Saini et al., 2013; Anwar et al., 2015).

The measurements of H^+^ flux in live *Y. lipolytica* cells showed the increase of the vanadate-sensitive H^+^ efflux in cells grown in the presence of Spm (Fig. 8), emphasizing a role of these signaling molecules in the regulation of H^+^ transport during the polarized growth of *Y. lipolytica*. A major advantage of the non-invasive system of selective ion electrode technique (SIET) is that acidification is directly probed very closely to the cell surface (∼10 µm), where local pH is usually quite different from that measured in the culture medium. In addition, the SIET data provides a real-time framework for the net H^+^ flux generated by the H^+^-ATPases across the plasma membrane, operating in live cells and detected as a balance between the H^+^ efflux driven by the pump and the H^+^ influx from the co-transport of solutes involved in cell nutrition as well as in building the osmotic pressure necessary for cell expansion.

In conclusion, this work provides compelling evidence for a mechanism by which hyphal growth is modulated by a Spm-dependent stimulation of P-type H^+^-ATPase, which in turn controls the hyphosphere pH, highlighting an unexplored developmental characteristic that fungi share with plants in the induction of cell polarized growth and morphogenesis towards multicellularity.

To the best of our knowledge, this is the first report describing a phytohormonal-like mechanism of action for fungal morphogenesis involving a polyamine-induced plasma membrane H^+^-ATPase activation in close correlation not only with the yeast-to-hypha transition but also with the colony structural dynamics. Since morphogenetic yeast-to-hypha transition and the polarized cell growth are key requirements for the ability of many fungi to invade, to adapt and survive under adverse conditions, this phenomenon has wide biological and biotechnological interest. In this context, such a mechanism constitutes a key target for interventions aiming to control the fungal production, pathogenesis and symbiosis for many industrial, medical and agronomic purposes.

## MATERIALS AND METHODS

### Yeast strain, media and cultivation conditions

The *Y. lipolytica* strain used in this work was JM12 (*MatB leu2-35 lys5-12 ura3-18*). The strain was kindly provided by Dr A. Dominguez (Universidad de Salamanca, Spain) and routinely maintained on YED medium (1% yeast extract, 1% glucose, 2% agar), supplemented with leucine, lysine, and uracil (each 50 mgl^−1^) adjusted to pH 4.5 with HCl. Liquid cultures were inoculated to an initial optical density at 600 nm (OD_600_) of 0.01 and incubated at 30°C at 250 rpm.

The polyamine spermine (Spm) stock solution (50 mM) was adjusted to a pH of 5.2 and filter-sterilized. Yeast cultures were supplemented or not with the Spm (1 mM) or IAA (10 pM), concomitantly or not with the auxin signaling inhibitor α-*p-*chlorophenoxy isobutyric acid (PCIB, 100 µM), the auxin efflux inhibitor 2,3,5-triiodobenzoic acid (TIBA, 100 µM), or the competitive polyamine biosynthesis inhibitor cyclohexylamine (CHA, 2 mM). All these compounds had no effect on *Y. lipolytica* cell growth. Cell morphology was analyzed at 2 h intervals using a Neubauer counting chamber in an Axio Imager A.2 microscope (Zeiss, Jena, Germany) and cell growth was monitored using an LGS53 spectrophotometer (Bel Photonics, Piracicaba, Brazil).

### Membrane isolation

Total membranes vesicles from *Y. lipolytica* cells were isolated as described previously (Okorokov and Lehle, 1998; Lobão et al., 2007) with some modifications. Briefly, the cells grown in YED medium pH 4.5 were transformed to the spheroplasts by incubation with lyticase from *Arthrobacter luteus* (Sigma-Aldrich, ≥200 units mg^−1^) at 37°C using 1.2 M sorbitol in 10 mM Tris-HCl, pH 7.2. Spheroplasts were homogenated in buffer containing 12.5% sucrose, 20 mM MOPS-Na, pH 7.6, 1 mM DTT, 1 mM benzamidine, 1 mM phenylmethanesulphonyl fluoride, a cocktail of protease inhibitors and 0.3% BSA, and total membranes were precipitated for 45 min at 100,000× ***g***. The total membranes were resuspended in buffer containing 12.5% sucrose, 20 mM MOPS-Na, pH 7.6, 1 mM DTT, 1 mM benzamidine, 1 mM phenylmethanesulphonyl fluoride and a cocktail of protease inhibitors, aliquoted and stored at −70°C.

### Plasma membrane H^+^-ATPase activity

To measure H^+^ transport, 30 µg of total membrane vesicles were added in incubation medium containing 20 mM MOPS-KOH, pH 7.2, 2.5 mM MgCl_2_, 50 mM KCl, 12.5% sucrose and 1 mM 9-amino-6-chloro-2-methoxyacridine (ACMA). H^+^ transport was initiated by addition of 1 mM ATP, pH 7.2, and monitored by the ﬂuorescence quenching of ACMA (Okorokov and Lichko, 1983) using a fluorescence spectrophotometer (RF5301PC, Shimadzu, Kyoto, Japan). Subsequent addition of 20 mM NH_4_Cl was used to show recovery of the ﬂuorescence that indicated a collapse of the preliminarily formed H^+^ gradient. F_max_ reﬂects steady-state amplitude of the ΔpH formation achieved after 10 min of H^+^ transport; it was calculated as ΔF/F and was expressed as a percentage. For determination of plasma membrane H^+^ transport, the membranes were pre-incubated with 0.2 mM Na_3_VO_4_, a speciﬁc inhibitor of P-type ATPase, before addition of ATP.

Initial velocity of H^+^ transport (*V*_0_) was determined by an extrapolation of the fluorescence quenching curve for 1 min. The plasma membrane ATP-dependent H^+^ transport was defined as the activity sensitive to pre-incubation with 0.2 mM sodium orthovanadate (Na_3_VO_4_), a specific inhibitor of P-type ATPase, and resistant to 110 nM concanamycin A, a specific inhibitor of V-H^+^-ATPase. ATPase activity was determined colorimetrically by measuring the release of Pi (Okorokov and Lichko, 1983; Fiske and Subbarow, 1925). The reaction media contained 30 mM MOPS-Tris, pH 6.5, 5 mM ATP, pH 7.2, 9.5 mM MgSO_4_ and 262 µM (NH_4_)_2_MoO_4_, with or without 0.2 mM Na_3_VO_4_. The reaction was started by addition of membrane vesicle protein and stopped with ice-cold 5% (w/v) trichloracetic acid after 30 min of incubation at 30°C. In all the experiments, the H^+^-ATPase activity was measured with and without vanadate, and the difference between these two activities was attributed to the plasma membrane H^+^-ATPase.

Protein concentrations in membrane preparations were determined by the method of Bradford (1976) using bovine serum albumin as standard.

### Western blotting

The immune reactivity of plasma membrane H^+^-ATPase was detected under conditions of low protein content to prevent a saturation of the cross-reactivity. Total membranes vesicles (10-20 µg) isolated from *Y. lipolytica* cells grown with or without 1 mM Spm for 14, 20, or 26 h were incubated at 65°C for 20 min, were separated on 10% (w/v) SDS-PAGE followed by an electrotransfer onto a nitrocellulose membrane (Hybond ECL, Amersham/GE Healthcare). H^+^-ATPase was detected using an anti-PMA1/PMA2 polyclonal antibody (1:1000) raised against the *S. cerevisiae* H^+^-ATPase from (Y-300; Santa Cruz Biotechnology). Immunodetection was performed using a peroxidase-conjugated anti-rabbit IgG secondary antibody (GE Healthcare Bio-Sciences) and with a chemiluminescence detection system kit (ECL Western blotting detection system, GE Healthcare). Intensities of immunoreactive bands on Western blots were quantified using ImageJ densitometric software (https://imagej.nih.gov/ij/).

### Plate-washing assay

The ability of *Y. lipolytica* cells for filamentous growth and agar invasion was determined as described (Cullen, 2015). Cell aliquots were spotted onto the surfaces of the YED plates containing or not 1.0 mM Spm and 2.0 mM CHA. The plates were incubated for 4 days at 30°C to allow the formation of colonies, and photographed. Plate surface was washed with water and photographed again.

### Scanning electron microscopy

To examine cell morphology in colonies, samples were prepared as described previously with modifications (França et al., 2011). *Y. lipolytica* cells were grown for 4 days at 30°C to allow the formation of colonies. The colonies were cut with agar from the plates and fixated with 2.5% glutaraldehyde, 4% formaldehyde in 0.1 M phosphate buffer (pH 7.2) for 24 h at a low temperature (5-6°C). Post-fixation was carried out for 2 h at room temperature with 1% osmium tetroxide. Initial dehydration was accomplished by placing colonies in the following series of ethanol gradients: 50% and 70% (two times for 10 min), 95% (two times for 5 min) and 100% (two times for 1 min), respectively. Then, samples were dehydrated with acetone (two times for 30 s). Samples were dried by the critical point method with CO_2_ in CPD-030 (BAL-TEC, Balzers, Liechtenstein). Subsequently, the colonies were coated with gold (20 nm) with sputter coater (SCD 050, BAL-TEC) and examined with a scanning electron microscope (DSM 962 EVO 40, Zeiss).

### Proton flux measurements

Proton flux was measured using non-invasive SIET essentially as described (Ramos et al., 2009). The ion-selective cocktails were from Sigma-Aldrich (Hydrogen ionophore I, Cocktail B, Cat. No.25293). *Y. lipolytica* cells were grown at 30°C for 16, 20 and 24 h in YED pH 4.5, with or without 1 mM Spm. The cells were collected by centrifugation, resuspended and immobilized on YED-agar and placed in a measuring chamber. Electrodes were positioned near the cell surface and the net H^+^ flux was measured for 10-15 min over an excursion distance of 15 µm as a µV difference. To analyze the H^+^ flux mediated by plasma membrane P-type H^+^-ATPase, 1 mM sodium orthovanadate was added to the cells and pre-incubated for 5 min prior to analysis. Control background measurements were performed at 500 to 700 µm distances from the cells, and subtracted from measurements performed near the cells surface.

### IAA determination

IAA was quantified with a Phytodetec-IAA immunodetection kit (Phytodetek^®^, Agdia Inc., Elkhart, USA) according to the manufacturer's instructions. *Y. lipolytica* cells were grown for 10, 14, 18, 22 or 26 h in YED medium at 30°C, pH 4.5, supplemented or not 1 mM Spm. The cells were centrifuged and the supernatant was collected and immediately methylated with (trimethylsilyl) diazomethane (Sigma-Aldrich, 1:1000).

### Statistical analyses

The data were analyzed using GraphPad Prism version 6.0 for Windows (GraphPad Software) and software package Origin version 8.0, and expressed as mean±s.d. All mean data were obtained from at least three independent experiments. The means were analyzed by ANOVA followed by Tukey test (*P*≤0.05). Statistically significant differences (*P*≤0.05) are indicated in the figures by different letters.
